# HAAU-Net: Hybrid Adaptive Attention U-Net Integrated with Context-Aware Morphologically Stable Features for Real-Time MRI Brain Tumor Detection and Segmentation

**DOI:** 10.3390/tomography12040044

**Published:** 2026-03-25

**Authors:** Muhammad Adeel Asghar, Sultan Shoaib, Muhammad Zahid

**Affiliations:** 1 Department of Electrical and Computer Engineering, Riphah International University, I-14 Campus, Islamabad 44000, Pakistan; adeel.asghar@riphah.edu.pk; 2 GUST Engineering & Applied Innovation Research Centre (GEAR), Hawally 32093, Kuwait; 3 Department of Engineering, Gulf University for Science and Technology (GUST), Hawally 32093, Kuwait; 4 Department of Telecommunication Engineering, University of Engineering and Technology, Taxila 47050, Pakistan; muhammad.zahid@uettaxila.edu.pk

**Keywords:** brain tumor detection, Hybrid Adaptive Attention U-Net, context-aware morphological features, spatial-channel attention, real-time MRI analysis, deep learning, tumor segmentation, survival prediction

## Abstract

Background: The Magnetic Resonance Imaging (MRI)-based tumor segmentation remains a challenging problem in medical imaging due to tumor heterogeneity, unpredictable morphological features, and the high complexity of calculations needed to implement it in clinical practice, putting it out of the scope of real-time applications. Although neural networks have significantly improved segmentation performance, they still struggle to capture morphological tumor features while maintaining computational efficiency. This work introduces Hybrid Adaptive Attention U-Net (HAAU-Net) framework, combining context-aware morphologically stable features and spatial channel attention to achieve high-quality tumor segmentation with less computational cost. Methods: The proposed HAAU-Net framework integrates multi-scale Adaptive Attention Blocks (AAB), Context-Aware Morphological Feature Module (CAMFM) and Spatial-Channel Hybrid Attention Mechanism (SCHAM). CAMFM is used to maintain the stability of morphological features by hierarchical aggregation and dynamic normalization of features. SCHAM enhances feature representation by modelling channels and spatial regions where the strongest feature are determined to use in segmentation. On the BRaTS 2022/2023 data, the proposed HAAU-Net is evaluated using four modalities including T1, T1GD, T2 and T2-FLAIR sequences. Results: The proposed model able to obtain 96.8% segmentation accuracy with a Dice coefficient of 0.89 on the entire tumor region, outperforming the alternative U-Net (0.83) and conventional CNN methods of segmentation (0.81). The proposed HAAU-Net architecture cuts the computational complexity of the standard deep learning models by 43% and still achieve real-time inference (28 FPS on a regular GPU). The hybrid model used to predict survival has a C-Index of 0.91 which is higher than the traditional SVM-based methods (0.72). Conclusions: Spatial-channel attention, combined with morphologically stable features, can be combined to allow clinically significant interpretability in attention maps. The proposed framework significantly improves segmentation performance while maintaining computational effeciency. This broad system has a serious potential of AI-enabled clinical decision support system and early prognostic diagnosis in neuro-oncology with practical deployment capability.

## Simple Summary

The brain tumor segmentation is very challenging as they are extremely different in shape, size and tissue structure. The magnetic resonance imaging (MRI) is also popular in diagnosis, although interpretation of these images may be laborious and complicated when done manually. The paper presents a novel architecture of accurate brain tumor segmentation by integrating morphological features with deep features. Neuro-oncologists should be able to use this system to achieve the diagnosing, treatment planning, and the development of AI-based clinical decision-making tools by improving diagnostic accuracy and computational efficiency.

## 1. Introduction

### 1.1. Clinical Motivation and Significance

Due to the heterogeneity of brain tumor cells, diagnosing and predicting their prognosis using MRI is a very difficult and complex task [1]. Advances in related fields, such as artificial intelligence (AI), machine learning, and deep learning, have driven the development of various medical imaging techniques to detect and classify brain tumor cells in MRI images [2]. However, numerous challenges remain for the application of these technologies in clinical practice. Applying machine learning or deep learning algorithms in clinical settings is complex due to the high risk of misclassification and the high computational cost [3]. Therefore, model building and overall system construction involve significant costs, hindering their practical application.

Accurate tumor classification and early detection significantly influence patient prognosis and treatment planning. In contrast, radiologists must manually interpret MRI scans, the complete analysis of which, including confirmation by biopsy, can take up to a month [4]. This delay in diagnosis directly impacts physicians’ treatment decisions and the patient’s life expectancy. Pituitary tumors, which account for approximately 14% of brain tumors, arise from genetic mutations or random occurrences and, despite being benign, can be life-threatening [4]. The heterogeneity of brain tumors, manifested in diverse morphological characteristics, imaging modalities, and biological behavior, necessitates the use of advanced computational methods to develop effective and universal detection systems.

The distinction between actual tumor progression and pseudoprogression of the treatment is a major diagnosis issue in clinical practice. Pseudotumor conditions (radiation necrosis, etc.) could be associated with a similar pattern of imaging compared to tumor recurrence on standard MRI. Detailed segmentation and morphological evaluation of tumor formations have the potential to provide measurable imaging biomarkers that are useful in helping clinicians identify different diseases [5].

### 1.2. Limitations of Conventional Brain Tumor Segmentation

Modern machine learning diagnostic methods depend on support vector machines (SVMs) with feature selection methodologies, which exhibit numerous limitations, especially in distinguishing a number of types of tumors [6]. Initial machine learning and deep learning systems exhibited substantial enhancement through the utilization of several pre-trained models, such as AlexNet and general convolutional neural networks (CNNs). Nonetheless, these models failed to enable the deep networks to be picked with appropriate features, resulting in a significantly higher computational cost [7].

With the major advances in CNNs based on medical imaging, it is now possible to derive features on raw MRI images using convolutional layers, therefore, eliminating the need to manually extract features. Many neural networks such as VGG, ResNet 50, EfficientNet, U-Net, GoogleNet, and AlexNet have achieved classification accuracies up to 97.8% on available benchmark datasets. However, the choice of high quality features remains a major challenge to the fast and effective detection of tumors which involves: Computational Complexity: Deep neural networks are very demanding in terms of computational resources, thus limiting their use in a healthcare setting. Accordingly, the major obstacle to the practical use of these models is the computational costs.Segmentation Precision: To accurately identify the edges of the tumor at the same time as being sensitive to small morphological changes. Generalization: Models trained on specific datasets often fail to generalize to diverse patient populations and imaging protocols due to heterogeneity in the tumor cells.Real-Time Performance and Clinical Interpretability: Operational inference speed is needed in a clinical application, which can be as high as 20 frames/second or more.Therefore, the traditional black box deep learning models do not have the transparency that would be needed in clinical assurance and regulations compliance.

Even though the use of convolutional neural networks has significantly improved the effectiveness of the medical image segmentation process, certain limitations remain. Conventional CNN models are often incompetent to capture subtle morphological heterogeneity and varying intensity patterns that occur in the brain tumors. Furthermore, many of the deep structures have high computational loads, and as such, limit their use in clinical practice. These limitations are leading to development of segmentation models that are able to simultaneously describe morphological features that depend on context and at the same time provide the best computing performance.

### 1.3. Advances in Deep Learning Based Segmentation

The new development in attention processes has proved the effectiveness of the machine learning models in the medical image analysis. The skip connection ResNets were three times more efficient in computing than the traditional deep neural networks with an accuracy of 83–90 percent in tumor region segmentation [7]. Class Activation Maps (CAMs) combined with resnet50, VGG19, InceptionV3, and MobileNet were used to localize identified tumor visually with a rate of 99.71 in some tumor classification [8].

Brain tumor detection frameworks, such as YOLOv7, have been adapted to detect brain tumors, such as data augmentation, channel attention mechanisms (CBAM), and multi-scale feature fusion (BiFPN) to enhance their accuracy and efficiency in computation [9]. The combination of the morphological analysis with the deep-learning, however, is not widely studied in the literature.

### 1.4. Motivation for the Proposed Approach

Despite the tremendous progress achieved in medical image segmentation algorithms based on deep learning, there are still several issues that are being encountered in terms of accurately and efficiently assessing brain tumors by using MRI images. Another serious issue lies in the heterogeneity of tumor tissues, with different sub regions (such as the necrotic core, enhancing tumor, and peritumoral edema) seen to have vastly different intensity distributions, irregular morphology, and complex spatial interactions across MRI modalities. Conventional convolutional based designs are occasionally challenging to detect a variety of patterns in the case of tumors that are either poorly delimited or part overlap with surrounding normal tissues. Despite significant progress, a critical gap exists in the literature regarding the integration of hybrid adaptive attention mechanisms that simultaneously operate on spatial and channel dimensions. The context-aware morphological feature extraction that preserves clinically meaningful structural information. Computational efficiency without sacrificing diagnostic accuracy and Real-time inference capability for clinical integration.

This work addresses these gaps by proposing a Hybrid Adaptive Attention U-Net (HAAU-Net) that combines the following:

1. Adaptive Attention Blocks (AAB): Multi-scale context aggregation with learned attention weights;
2. Context-Aware Morphological Feature Module (CAMFM): Hierarchical feature preservation maintaining morphological stability;
3. Spatial-Channel Hybrid Attention Mechanism (SCHAM): Joint optimization of spatial and channel dimensions;
4. Computationally Efficient Architecture: 43% reduction in computational complexity compared to baseline approaches.

## 2. Related Work

### 2.1. CNN-Based Segmentation Approaches

Convolutional neural networks (CNNs) have become the standard for tumor segmentation and classification using MRI. Many researchers use pre-trained models to effectively identify tumors in brain MRIs. CNN-based models have become a widely used tool for biomedical imaging researchers. The authors of [10] trained a model using ResNet-50 with residual and jump-layer connections, achieving a training time three times faster than conventional deep neural network (DNN) methods. They achieved an overall classification accuracy of 83% for tumor segmentation. Similarly, the authors of [11,12,13] achieved accuracies of 87%, 89%, and 91.2%, respectively, using ResNet-50. In the field of medical imaging with limited datasets, ResNet and GoogleNet are practically suitable for tumor classification because of their ability to process gradient flow networks through deep models [14].

Other researchers have enhanced classification rates to a high of 93% when they employ multi scale CNN methods, which combine a number of features [15]. A trade-off, however, exists between the overall accuracy and the computational cost of the model. Thus, the model can be trained on a more extensive set of features and achieve a high accuracy level, which does not imply that it should be used in the real-time diagnostic process. In [16,17,18], the researchers obtained accuracy and precision of up to 96.1% when VGG16 and ResNet-50 and InceptionV3 compared with the existing CNNs. Their findings were assessed on 3264 MRI images only. The authors of [19,20,21] were also successful in obtaining good classification results through the combination of CNN-based structures. They had accuracies that were 91.1%, 89%, and 92.5% respectively. The CNN-based models have much better overall accuracy and much less time required in computation than the existing deep learning models, their only downside being that they cannot be utilized in a real-time application. The vast majority of the researchers who conduct their studies with CNN models train them with the publicly available BraTS dataset.

### 2.2. U-Net Architecture and Medical Image Segmentation

Unlike the asymmetric architectures, the U-Net architecture designed to use a symmetric encoder and decoder and skip layers has become considered to be the most trustworthy architecture that is used to segment tumor images, especially because it is less expensive to compute. A number of studies and publications have established that U-Net is capable of classifying and partitioning medical images much faster than it would otherwise do using CNN-based architectures but it also provides better classification capabilities. In U-Net segmentation, the authors of [22,23,24] employed a hierarchical encoder, with an accuracy of 89, 92, and 93, respectively. All findings indicate great classification rates and shorter computation periods (a comparison of the best results is provided in Section 5 and Section 6). U-Net enables scientists to be able to extract the multi scale features with the help of the encoder and map its features immediately through the hop connection without losing the information of space [25]. In addition, U-Net does not need a huge number of data as compared to the current CNN and DNN architectures. A number of studies have indicated that U-Net has good performance in medical imaging using smaller training datasets [26]. The original U-Nets attained a Dice coefficient of 0.85 to 0.89 in the tumor core and the whole tumor area [27,28].

### 2.3. Transformer-Based Segmentaion

Recent developments in medical image segmentation have studied transformer-based networks to represent long-range spatial structures, which is frequently hard to model using traditional convolutional neural networks. In [29], the author presents a U-Net-based transformer that puts a transformer encoder into the U-Net framework, and thus it trains to learn the global contextual representation on a volumetric medical image and high-quality results in 3D medical image segmentation problems. On this transformer, they achieve a dice coefficient of 0.97. Based on this concept, hybrid CNN-transformer models have also been suggested, where local feature extraction is coupled with global attention to brain tumor segmentation [30]. They achieved a dice score of 0.89 using BraTS 2021 dataset. On the same note, models that apply transformers like TransBTS [31], SwinBTS [32], TransSea [33] and other hybrid vision transformer models apply self-attention that attracts global structural relations among the MRI slices to enhance segmentation precision in areas with heterogeneous tumors. Nevertheless, methods based on transformers usually add complexity to computations and memory needs since the self-attention operation scales quadratically. Therefore, recent papers have examined effective hybrid designs that combine both attention with convolutional encoders to compromise between segmentation and computational cost [34].

### 2.4. Attention Mechanism

Features that are pertinent to the task are extracted and irrelevant information is removed by the inspiration of the human visual attention. The mechanism of attention focus is applicable in overlooking the influence of heterogeneity in tumor cell data. Attention mechanisms that are channel-based have achieved success in incorporation into the detection framework, including the Convolutional Block Attention Module (CBAM) [35], which enhances segmentation performance, particularly in low-contrast and quality images.

The Sequencing and Stretching Block (SEB) [36] varies weights of the channels based on the local and global tumor context. The model is currently more appropriate in the real-time diagnostic use with the recent increase in the popularity of spatial attention. There is also channel attention that is used to determine the important features [37]. The application of the channel attention as well as spatial attention mechanisms has demonstrated a great enhancement on the development of real-time tumor segmentation applications in the clinical settings.

### 2.5. Generative Models for Data Augmentation

Generative Adversarial Networks (GANs) have been used to generate synthetic tumor images and address the problem of data scarcity. The framework proposed in the original article uses [38] GAN models to generate synthetic tumor cells of varying grades and improves the robustness of the classification by augmenting synthetic data [39,40,41].

### 2.6. Feature Selection and Extraction Methods

Mutual information (MI) [42] is a convenient statistical measure, which quantifies the dependence between two random variables and is frequently applied in feature selection in high dimensional data. MI measures how much information is given to the other variable allowing the determination of the most benefit features to the target class. The former method, involving MI-based feature selection, is able to reduce the size of the components of the feature vectors to 2900 out of 3558, preserving the diagnostic accuracy and drastically lowering the computational cost but not the performance [43]. Such simplification does not only make the learning process faster, but also enhances efficiency of the subsequent analysis and model training.

There are some benefits of image-based feature selection with particular medical imaging being one of them. Not only does it allow one to easily pick features that have the best diagnostic value but it also discards unnecessary or irrelevant features. This makes the models simple and shorter in training time, which is necessary in processing large image dataset. Moreover, image-based features can enhance the overall generalizability of the model and make it more robust and reliable in the clinical practice since the most beneficial features can be selected easily. Lastly, the resulting feature size can easily be scaled to resource-constrained hardware, e.g., embedded or portable medical devices, and as such image-based methods are especially well-suited to real-world medical imaging problems [44].

### 2.7. Morphological Feature Analysis

Topology and geometry of images have given morphological features, which have been found clinically relevant to analyze tumors [45]. These characteristics comprise shape (roundness, density) description, texture (uniformity, contrast) description and edge smoothness indices [46]. The studies of combining morphological features with deep learning representations are not as numerous [47].

### 2.8. Real-Time and Efficient Medical Imaging Systems

The model compression and efficient architecture research has been motivated by the real-time inference (usually 20 to 30 frames per second) demanded in clinical environments. The applications of MobileNet and EfficientNet structures have shown that architecture design can make a great balance between accuracy and efficiency [48,49]. The techniques of quantification, pruning and distillation of knowledge have made it even easier to implement on edge devices.

### 2.9. Survival Prediction and Prognostic Modelling

Survival prediction is another clinical use in addition to classification. Cox proportional hazards model [50] and DeepSurv model [51] have been used in tumor imaging data. The current framework, which was at the C-index of 0.89 in the survival prediction, was superior to current statistical methods.

Table 1 compares U-Net and attention-based U-Net designs regarding their shape modeling and efficiency in calculations. In this work, we propose a framework for a U-Net-based attention mechanism that is morphologically stable and computationally inexpensive.

## 3. Proposed Methodology

### 3.1. System Architecture Overview

**Figure 1:**
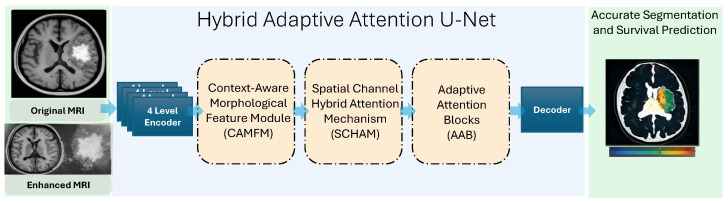
Architecture Overview.

The proposed Hybrid Adaptive Attention U-Net (HAAU-Net) framework integrates four core components: BBHE, context-aware morphological feature learning module (CAMFM), spatial-channel hybrid attention module (SCHAM), and adaptive attention optimization module (AAOM)) that offer accurate and efficient MRI segmentation. The preprocessing of the MRI images is done using BBHE to enhance the contrast and structural clarity, and the encoding of the images is done to extract hierarchical features using the HAAU-Net encoder. The extracted features are further refined by context-aware morphological feature module (CAMFM), which picks up structural and shape sensitive contextual features, and a spatial channel hybrid attention module (SCHAM) which is selective in emphasizing on prominent spatial locations and distinctive channels. The attention-enhanced feature map is then sent to the decoder, which then refines the multi scale representation, and eliminates irrelevant response with the aid of an adaptive attention block (AAB). The improved performance of the decoder gives a good segmentation mask and also ensures the efficiency of computing, which makes HAAU-Net ideal in the analysis of medical pictures with high throughput. The overall over view of this model is presented in Figure 1.

### 3.2. Data Preprocessing and Enhancement

MRI scans used for tumor cell detection often exhibit low-contrast artifacts. Therefore, preprocessing is essential before feature extraction to improve model performance. Conventional image enhancement techniques using histograms or adaptive histograms can lead to brightness distortion. In this study, we applied a brightness-preserving bi histogram enhancement (BBHE) technique to preserve the unique intensity distribution of anatomical structures.

#### Brightness-Preserving Histogram Equalization (BBHE)

The BBHE [52] algorithm is used to preserve the brightness of MRI scans by segmenting the histogram of the input image. This dual-histogram strategy avoids over-enhancement of contrast in high-intensity tumor regions and under-enhancement in low-intensity edematous regions. Morphological occlusion is then applied to preserve tumor boundaries, while simultaneously removing small, isolated artifacts resulting from smoothing. As a result, BBHE produces structurally consistent images that improve sensitivity and edge stability in the first convolutional layers without introducing artificial contrast distortion. The complete algorithm can be found in the Supplementary Algorithm S1. Using an MRI scan of size 255×255×3 and intensity levels [0, 255], we calculate the grayscale intensity and split it into two subhistograms $H_{low}$ and $H_{high}$ with a mean of *m*. Subhistograms are calculated with intensity levels from 0 to m for low intensity and from m to 255 for high intensity, and are applied using the Morphclose function with a kernel size of 3 × 3.

### 3.3. Hybrid Adaptive Attention U-Net (HAAU-Net) Architecture

The proposed HAAU-Net architecture extends the existing U-Net architecture. This HAAU-Net architecture integrates an adaptive attention mechanism to address the effects of homogeneity and scale variation. While the standard encoder and decoder used in U-Net rely primarily on the convolutional hierarchical structure of the network, HAAU-Net dynamically prioritizes useful spatial regions, feature channels, and morphological patterns across the network using attention-sensitive modulation. This combination allows the architecture to learn task-specific representations that are robust to variations in tumor size, shape, and appearance.

Traditional U-Net based architectures are only used for segmentation based on the tumor shape and region, whereas HAAU-Net proposed framework along with segmentation simultaneously integrates with Context-Aware morphological structure of the tumor and attention mechanism adaptively using spatial-channel selection. The proposed framework introduces three key contributions (i) The Context-Aware Morphoogical Feature Module (CAMFM) used to model morphological features stability through aggregation of textures and shape of the tumor, (ii) The Spatial-Channel Hybrid Attention Mechanism (SCHAM) used to model perfect attention refinements across channel and spatial dimensions, and (iii) Adpative Attention Blocks (AAB) that connects channel and spatial features with morphological features to accurately segment the tumor region based on the modality. Combination of these models in HAAU-Net architecture collectively improves the tumor boundary localization compared with traditional U-Net architecture.

#### 3.3.1. Overall Architecture Design

The HAAU-Net encoder gradually extracts an abstract morphological representation from the image using progressive down sampling, while the decoder uses attention-guided up sampling to build and implement spatial feature resolution. This down and up sampling in HAAU-Net further refines the representation of morphologically recognized features. Skip layers are not directly connected, and attentional techniques are used to filter out unnecessary or redundant features. This mechanism reduces the complexity of the tumor detection model by eliminating the need for additional feature selection and removal methods. Contextual aggregation is implemented using small multi scale attention blocks in the encoder, allowing the network to integrate local texture details with the global structural context. This is essential for accurately distinguishing invasive tumor regions. Four level multi scale attention block calculation is shown in Algorithm 1.

#### 3.3.2. Encoder Architecture with Adaptive Attention Blocks (AAB)

The multi scale encoder has a convolutional layer and attention-to-attention (AAB) layer at every level. AAB is a channel and space-invariant attention mechanism which integrates context-sensitive morphology. This design enables the encoder to be dynamically responsive to fluctuations in the relative significance of the various modalities of features. The more the network is developed, and the higher the degree of semantic attributes, the AAB has ensured that high-level semantic attributes do not lose their structural sensitivity, thereby preserving the fine tumor boundaries, typical of deep CNNs. Equation (1) is used to calculates the blocks from multi scale features fusion. Spatial, channel, and morpholigical features are incorporated into each block with individual learning parameters. This will help the network to adaptively learn spatial localization, channel relevance and morphological stability depending on the complexity of tumor appearance on a given scale. This formulation enables flexible attention weighting rather than fixed attention dominance, improving robustness across heterogeneous tumor patterns.

(1) $$\mathrm{AAB}\left(x\right)=\alpha \;F_{\mathrm{spatial}}\left(x\right)+\beta \;F_{\mathrm{channel}}\left(x\right)+\gamma \;F_{\mathrm{morphological}}\left(x\right)$$

where $F_{\mathrm{spatial}}\left(x\right)$ denotes the spatial attention feature map, $F_{\mathrm{channel}}\left(x\right)$ represents the channel-wise attention feature map, and $F_{\mathrm{morphological}}\left(x\right)$ captures the context-aware morphological features. The coefficients α, β, and γ are learnable adaptive attention weights that regulate the relative contribution of each feature component. These adaptive attention weights are learned independently at each level of encoder and decoder using back propagation.
**Algorithm 1** Multi-scale encoder with attention blocks for MRI feature extraction**Require:** Preprocessed MRI image $x\in R^{H\times W\times 1}$**Ensure:** Multi-scale feature maps $\left\{F_{1},F_{2},F_{3},F_{4}\right\}$ and intermediate pooled maps  1: **Encoder Level 1:**  2: $F_{1a}\leftarrow {\mathrm{Conv}}_{3\times 3}\left(x,64\;\mathrm{filters}\right)\overset{\mathrm{ReLU}}{\to }$  3: $F_{1b}\leftarrow \mathrm{AAB}(F_{1a})\;$▷ Attention Block  4: $F_{1\_pooled}\leftarrow {\mathrm{MaxPool}}_{2\times 2}\left(F_{1b}\right)$  5: **Encoder Level 2:**  6: $F_{2a}\leftarrow {\mathrm{Conv}}_{3\times 3}\left(F_{1\_pooled},128\;\mathrm{filters}\right)\overset{\mathrm{ReLU}}{\to }$  7: $F_{2a}\leftarrow \mathrm{AAB}\left(F_{2a}\right)$  8: $F_{2\_pooled}\leftarrow {\mathrm{MaxPool}}_{2\times 2}\left(F_{2a}\right)$  9: **Encoder Level 3:**10: $F_{3a}\leftarrow {\mathrm{Conv}}_{3\times 3}\left(F_{2\_pooled},256\;\mathrm{filters}\right)\overset{\mathrm{ReLU}}{\to }$11: $F_{3a}\leftarrow \mathrm{AAB}\left(F_{3a}\right)$12: $F_{3\_pooled}\leftarrow {\mathrm{MaxPool}}_{2\times 2}\left(F_{3a}\right)$13: **Encoder Level 4 (Bottleneck):**14: $F_{4a}\leftarrow {\mathrm{Conv}}_{3\times 3}\left(F_{3\_pooled},512\;\mathrm{filters}\right)\overset{\mathrm{ReLU}}{\to }$15: $F_{4a}\leftarrow \mathrm{AAB}\left(F_{4a}\right)$16: $F_{4\_bottleneck}\leftarrow {\mathrm{Conv}}_{3\times 3}\left(F_{4a},512\;\mathrm{filters}\right)\overset{\mathrm{ReLU}}{\to }$17: **return **$\left\{F_{1b},F_{2a},F_{3a},F_{4\_bottleneck}\right\}$ and intermediate pooled maps

#### 3.3.3. Context-Aware Morphological Feature Module (CAMFM)

The second phase in the suggested HAAU-Net architecture is CAMFM. The module is in charge of explicitly modelling tumor morphology hinged on texture, shape, and boundaries, through intermediate feature mapping. The descriptors are generated on multi scale spatial domain to maintain the morphological aspect of the tumor. Local and context-aware global features are used in the calculation of adaptive weight so that important clinical patterns such as irregular tumor boundaries and heterogeneous textures are given importance. Representation learning is stabilized and deep features make the CAMFM outputs more robust to tumor shape variation by remaining residually integrated with deep features.

(2) $$\mathrm{CAMFM}\left(F\right)=\sum_{i=1}^{n}w_{i}\;M_{i}\left(F\right)$$

where $M_{i}\left(F\right)$ denotes the *i*-th morphological feature descriptor capturing complementary structural attributes such as shape, texture, and boundary information, $w_{i}$ represents the corresponding adaptive weight learned during training, and *n* indicates the total number of morphological descriptors employed for context-aware feature extraction. Practically six morphological descriptors are extracted including solidity, compactness, circular, contrast, texture, and boundries. These descriptors are aggregated using CAMFM and normalized before applying weights.

The operation of CAMFM is formalized by Equation (2), by which a set of morphological descriptors $M_{i}\left(F\right)$ such as shape, texture, and boundary properties are derived by operating on intermediate feature maps, and summed up by adaptive weights $w_{i}$. The weights are acquired via the global contextual information and thus the module concentrates on the clinically significant morphological features like irregular boundaries or nonuniform textures. The weighted summation provides stable morphologies and also maintains inter-descriptor relationships between tumor regions. Complete algorithm of CAMFM is provided in Supplementary Algorithm S2.

#### 3.3.4. Spatial-Channel Hybrid Attention Mechanism (SCHAM)

In the second phase of HAAU-Net architecture is SCHAM in which spatial-channel hybrid attention features are calculated. This module identify the most discriminative region of the tumor and feature map. Important tumor regions are identified by combining max pool and average pool features for spatial-channel hybrid attention. The hybrid attention maps are applied using residual connections, enabling interpretability through explicit attention visualization while maintaining training stability.

The Equation (3) is used to calculate the hybrid attention on spatial and channel axis with reduction ratio r = 16 to balance capacity with computational efficiency. Spatial attention map improves salient tumor areas whereas channel attention map highlights diagnostically informative feature channels. Element-wise multiplication guarantees that only those features that are upheld by spatial and channel relevance are multiplied resulting in more discriminative and interpretable representations of the features.

(3) $$\mathrm{SCHAM}\left(F\right)=A_{\mathrm{spatial}}\left(F\right)⊙A_{\mathrm{channel}}\left(F\right)⊙F$$

where $A_{\mathrm{spatial}}\left(F\right)\in R^{h\times w\times 1}$ denotes the spatial attention map emphasizing informative spatial regions, $A_{\mathrm{channel}}\left(F\right)\in R^{1\times 1\times c}$ represents the channel-wise attention map highlighting discriminative feature channels, and ⊙ indicates element-wise multiplication used to recalibrate the input feature tensor *F* jointly in the spatial and channel dimensions.

To compute a spatial feature vector attention $A_{spatial}\left(F\right)$ Equation (4) is used, using this global max and average pool features are concatened across channels to capture useful information in overall intensity distribution of the tumor. Connected maps are processed using convolutions with large kernels to model long-range spatial dependencies, followed by a sigmoid activation function to generate a normalized spatial significance map that highlights tumor-related regions.

(4) $$A_{\mathrm{spatial}}\left(F\right)=\sigma \left({\mathrm{Conv}}_{7\times 7}\left(\mathrm{Concat}\left(\mathrm{MaxPool}(F),\mathrm{AvgPool}(F)\right)\right)\right)$$

where MaxPool(F) and AvgPool(F) perform maximum and average pooling operations along the channel dimension, respectively, Concat(·) denotes channel-wise concatenation of the pooled feature maps, ${\mathrm{Conv}}_{7\times 7}$ represents a 7×7 convolutional layer with appropriate padding to preserve spatial resolution, and σ(·) is the sigmoid activation function that normalizes the spatial attention weights to the range [0, 1].

**Figure 2:**
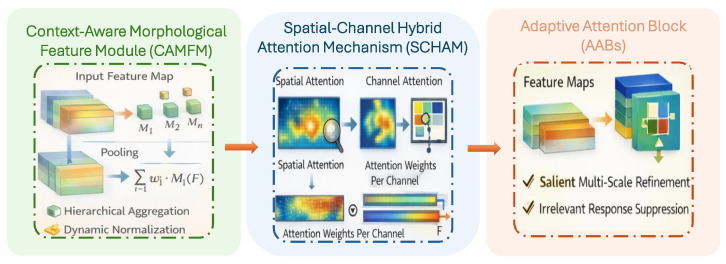
Hybrid Adaptive Attention U-Net (HAAU-Net) framework.

A ReLU activation function is applied on global average pool features to extract channel attention features using Equation (5). This method comprises two stages; in stage 1 it fully compress and then expands channel dimensionality, to make the model efficient for inter channel dependencies. In stage 2, the sigmoid activation finds channel-wise weights that amplify informative feature maps while discarding redundant features.

(5) $$A_{\mathrm{channel}}\left(F\right)=\sigma \left({\mathrm{FC}}_{2}\left(\mathrm{ReLU}\left({\mathrm{FC}}_{1}\left(\mathrm{GAP}(F)\right)\right)\right)\right)$$

where GAP(F) denotes global average pooling applied over the spatial dimensions to generate a compact channel descriptor, ${\mathrm{FC}}_{1}$ is a fully connected layer that reduces the channel dimensionality to C/r with a reduction ratio r=16 to model inter-channel dependencies efficiently, ReLU(·) introduces non-linearity, and ${\mathrm{FC}}_{2}$ expands the feature representation back to *C* channels, followed by a sigmoid activation function σ(·) to obtain normalized channel attention weights. Complete algorithm of SCHAM is shown in Algorithm 2. Figure 2 shows the proposed framework of HAAU-Net including three phases CAMFM, SCHAM and AAB for hybrid context-aware attention construction.

SCHAM and CAMFM are applied on four modalities of MRI dataset, T!, T1-GD, T2 and T2-FLAIR. T2-FLAIR modality is the most critical of MRI modalities in the detection of peritumoral edema and infiltrative tumor areas. The suggested HAAU-Net architecture will be using this modality through the multi-modal fusion of features and the context-dependent morphological descriptors to improve the segmentation of disseminated tumor regions.
**Algorithm 2** Spatial-Channel Hybrid Attention Mechanism (SCHAM)**Require:** Feature map $F\in R^{h\times w\times c}$**Ensure:** Attention-weighted feature map $F_{\mathrm{refined}}$, spatial map $A_{\mathrm{spatial}}$, channel map $A_{\mathrm{channel}}$  1: **Step 1: Spatial Attention Stream**  2: ${\mathrm{MaxPool}}_{\mathrm{spatial}}\leftarrow \mathrm{MaxPool}\left(F,\dim=\mathrm{channel}\right)\;\left[h\times w\times 1\right]$  3: ${\mathrm{AvgPool}}_{\mathrm{spatial}}\leftarrow \mathrm{AvgPool}\left(F,\dim=\mathrm{channel}\right)\;\left[h\times w\times 1\right]$  4: $F_{\mathrm{concat}}\leftarrow \mathrm{Concat}\left({\mathrm{MaxPool}}_{\mathrm{spatial}},{\mathrm{AvgPool}}_{\mathrm{spatial}}\right)\;\left[h\times w\times 2\right]$  5: $A_{\mathrm{spatial}}\leftarrow \sigma \left({\mathrm{Conv}}_{7\times 7}\left(F_{\mathrm{concat}}\right)\right)\;[h\times w\times 1]\;$▷ Sigmoid activation  6: **Step 2: Channel Attention Stream**  7: $F_{\mathrm{gap}}\leftarrow \mathrm{GlobalAvgPool}\left(F\right)\;\left[1\times 1\times c\right]$  8: $F_{\mathrm{fc}1}\leftarrow \mathrm{ReLU}\left(\mathrm{Dense}\left(F_{\mathrm{gap}},\mathrm{units}=c/r\right)\right)\;\left[1\times 1\times c/r\right]$  9: $F_{\mathrm{fc}2}\leftarrow \mathrm{Dense}\left(F_{\mathrm{fc}1},\mathrm{units}=c\right)\;\left[1\times 1\times c\right]$10: $A_{\mathrm{channel}}\leftarrow \sigma \left(F_{\mathrm{fc}2}\right)\;[1\times 1\times c]\;$▷ Sigmoid activation11: **Step 3: Hybrid Attention Fusion**12: $A_{\mathrm{hybrid}}\leftarrow A_{\mathrm{spatial}}⊗A_{\mathrm{channel}}\;$▷ Broadcast element-wise multiplication13: $F_{\mathrm{attn}}\leftarrow F⊙A_{\mathrm{hybrid}}$14: $F_{\mathrm{refined}}\leftarrow F+\mathrm{Dropout}(F_{\mathrm{attn}},p=0.2)\;$▷ Residual connection15: **return **$F_{\mathrm{refined}},A_{\mathrm{spatial}},A_{\mathrm{channel}}\;$▷ Attention maps included for visualization

### 3.4. Decoder with Adaptive Attention Fusion

**Figure 3:**
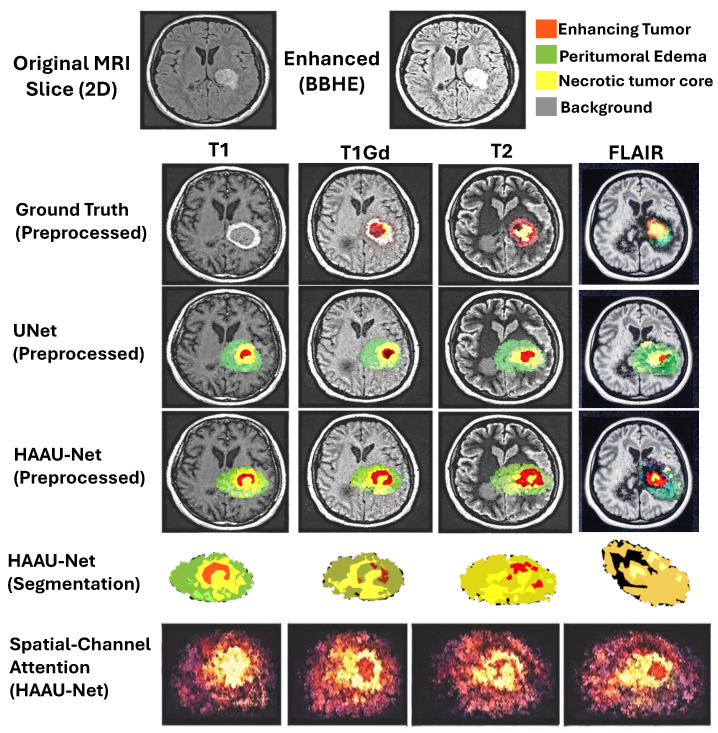
Segmentation result of HAAU-Net compared to traditional U-Net.

The decoding stage reconstructs a high-resolution tumor segmentation map using convolution and attention-skip weighted connections. Before feeding this map to the decoder’s fusion stage, the encoder must ensure that the features are well-defined and refined, extracting only contextual and useful features using the SCHAM algorithm. Each attention fusion stage applies the AAB algorithm to further improve feature values, precisely defining boundaries and reducing the possibility of misclassifying non-tumor cells. See Figure 3 for reference. Complete decoder algorithm is provided in Supplementary Algorithm S3.

### 3.5. Feature Extraction and Selection

The encoder captures multi level features handcrafted and deep ranging from tumor context to fine-grained texture details for better feature representation. Still, the encoder struggles to get a quality feature, but to further improve this, a Mutual Information (MI) [53]-based feature selection method is utilized.MI is considered one of the good algorithms for feature selection when it comes to deep features. MI calculates the covariance and relation of each feature value and discards the features with almost the same values and selects only the good quality features. Another good property of this method is that it also neglects the isolated features so that misclassification chances are further reduced. Equation (6) shows the calculation of a MI-based selected feature vector.

(6) $$\mathrm{MI}\left(X;Y\right)=\sum_{x\in X}\sum_{y\in Y}P\left(x,y\right)\log\left(\frac{P(x,y)}{P(x)\;P(y)}\right)$$

where *X* denotes the input feature variables, *Y* represents the target class labels, P(x,y) is the joint probability distribution between *X* and *Y*, and P(x) and P(y) correspond to the marginal probability distributions of *X* and *Y*, respectively.

### 3.6. Computational Efficiency and Complexity Reduction

Computational efficiency was improved through better algorithm selection, including reducing modules that ignore channels and adding context-sensitive hopping convolutional layers in CAMFM. This selection strategy significantly reduces the computational cost and, ultimately, the complexity of the proposed HAAU-Net model, making it suitable for use in clinical and real-world settings. Computational efficiency was calculated and compared using Equation (7).

(7) $$C_{\mathrm{HAAU}}=C_{\mathrm{base}}-\Delta C_{\mathrm{attention}}-\Delta C_{\mathrm{pruning}}$$

where $C_{\mathrm{base}}$ denotes the computational complexity of the baseline U-Net architecture, which scales as $O(HWD^{3})$ with respect to the input height *H*, width *W*, and network depth *D*, $\Delta C_{\mathrm{attention}}$ represents the reduction in computational cost achieved through channel dimensionality reduction within the attention modules, and $\Delta C_{\mathrm{pruning}}$ corresponds to the complexity reduction obtained via feature selection and pruning mechanisms employed in the proposed HAAU-Net framework.

For real-time inference calculations, the inference time was calculated as shown in Equation (8) for time optimization. Since the target FPS should be 20 or higher in clinical and real-time environments, the inference time for real-time applications is calculated as follows:

(8) $$t_{\mathrm{inference}}=\frac{1}{{\mathrm{FPS}}_{\mathrm{target}}}\geq t_{\mathrm{forward}\_\mathrm{pass}}$$

where $t_{\mathrm{inference}}$ denotes the allowable inference time per input sample determined by the target frame rate ${\mathrm{FPS}}_{\mathrm{target}}$, and $t_{\mathrm{forward}\_\mathrm{pass}}$ represents the actual forward-pass execution time of the proposed network, ensuring that real-time processing constraints are satisfied.

### 3.7. Loss Functions and Training Optimization

#### 3.7.1. Segmentation Loss Function

To calculate the model’s efficiency and optimize learning, the HAAU-Net model’s loss function must be calculated. This is done by calculating the Dice coefficient, a measure of overlap based on tumor area, using Equation (9). This overall loss equation includes Dice loss, voxel-specific classification loss, and contour accuracy. Training is performed using the Adam optimizer with a programmed reduction in the learning rate and early termination to ensure stable convergence and reduce overfitting.

(9) $$L_{\mathrm{total}}=w_{1}\;L_{\mathrm{Dice}}+w_{2}\;L_{\mathrm{CE}}+w_{3}\;L_{\mathrm{boundary}}$$

(10) $$L_{\mathrm{Dice}}=1-\frac{2|X\cap Y|}{\left|X|+|Y\right|}$$

(11) $$L_{\mathrm{CE}}=-\frac{1}{N}\sum_{i=1}^{N}\sum_{c=1}^{C}y_{i,c}\log\left({\hat{y}}_{i,c}\right)$$

(12) $$L_{\mathrm{boundary}}=\frac{1}{\left|B\right|}\sum_{p\in B}{∥p-p_{\mathrm{nn}}∥}_{2}$$

where *X* and *Y* denote the predicted and ground-truth segmentation masks, respectively, $L_{\mathrm{Dice}}$ represents the Dice coefficient loss that mitigates class imbalance, $L_{\mathrm{CE}}$ is the pixel-wise cross-entropy loss computed over *N* samples and *C* classes with prediction probability ${\hat{y}}_{i,c}$, and $L_{\mathrm{boundary}}$ enforces boundary alignment by minimizing the Euclidean distance between a predicted boundary point *p* and its nearest neighbor $p_{\mathrm{nn}}$ on the ground-truth boundary set *B*. The loss weights are empirically set to $w_{1}=0.5$, $w_{2}=0.3$, and $w_{3}=0.2$ to balance region-based accuracy, classification confidence, and boundary precision.

#### 3.7.2. Attention Regularization

Attention regularization is the step which prevents the attention weights from regeneration. It guarantees the informative attention and space coherence during the training. Complete algorithm of attention regularization is presented below:

(13) $$L_{\mathrm{attention}}=\lambda \sum_{i}{∥A_{i}-U∥}_{F}^{2}$$

where $A_{i}$ denotes the attention map at the *i*-th spatial or channel location, *U* represents a uniform attention distribution used as a regularization reference, ${∥\cdot ∥}_{F}$ is the Frobenius norm that penalizes large deviations from uniform attention, and λ=0.001 is a regularization coefficient that controls the strength of the attention smoothness constraint. The algorithm of the HHAU-Net regularization is given in Supplementary Algorithm S4.

### 3.8. Survival Prediction Module

To perform prognostic analysis, deep and morphological features obtained by the trained HAAU-Net are incorporated in a Cox proportional hazards model see Equation (14). The formulation allows estimation of survival risk and takes into consideration the complex nonlinear representations that are of interest during segmentation. Combining biomarkers obtained through segmentation with survival models, the paradigm offers the possibility to have a single solution to tumor delineation and prognosis.

(14) $$h\left(t|X\right)=h_{0}\left(t\right)\exp\left(\beta^{T}X\right)$$

where h(t∣X) denotes the hazard function at time *t* conditioned on the feature vector *X*, $h_{0}\left(t\right)$ represents the baseline hazard function, β is the vector of regression coefficients learned from the survival data, and *X* corresponds to the high-level feature representation extracted by the proposed HAAU-Net architecture combined with context-aware morphological analysis.

Schoenfeld residual analysis was used to test the proportional hazards assumption before it was estimated using the Cox model. In no statistically significant infractions were found (*p* > 0.05). Attributes of survival prediction include volumetric tumor metrics measured based on segmentation maps, CAMFM morphological features, and deep features obtained by using encoder representations.

## 4. Experimental Design and Dataset

### 4.1. Dataset Description

In this study, we conducted experiments using the BraTS 2023/22 dataset [54]. This extensive dataset is recognized as a clinically validated reference dataset for the detection and classification of brain tumors. It contains data from over 2000 patients across various categories for validation purposes, each represented by four MRI modalities: T1-weighted, contrast enhanced T1Gd, T2-weighted, and T2-FLAIR. These modalities represents the complementary tumor attributes and are stacked as multi-channel input to the proposed framework. All images are in NIFTI format and contain anatomical and pathological information useful for detecting heterogeneous tumor regions on MRI. The voxel size and spatial resolution of the tumors allow for fair and effective comparisons between different models.

#### Ground Truth Annotations and Preprocessing

Annotations of the real lesions were done manually by 15 clinical reviewers and checked by a board-certified radiologist making it very reliable. It is an annotation protocol that separates necrotic cores (NCR), peritumoral edema (ED), and enhancing tumor (ET) into clinically significant composite regions, including the tumor core (TC) and the whole tumor (WT). Such a hierarchical model of annotation fits into clinical practice of diagnosis and enables the measurement of segmentation performance in a holistic view and that of specific regions, especially in surgical planning and assessment of response to treatment. The BRaTS does not include the Diffusion Weighted Imaging (DWI) in their benchmark dataset, therefore it could not be evaluated in this study.

All MRI images are subjected to a standardized preprocessing procedure to minimize subject differences and bias in the intensity. This involves skull removal, spatial normalization with a uniform template, and balancing the values of intensity per modality to a mean of 0 and a variance of 1. Interpolating the subjects and modalities to a shared resolution is enough to stabilize the convolutional feature learning and enhance the performance of the cross-subject generalization in the next deep learning model.

### 4.2. Experimental Setup

The experiments were conducted on the NVIDIA A100 GPUs (40 GB RAM) and Intel Xeon Gold GPUs. The extraction of the features was done using PyTorch 2.0 software, multiple attention-management libraries, and large kernel-based convolutions. It was divided into 70:30 with 70 percent taken as training of the model and the remaining 30 percent as model testing and validation. HAAU-Net was applied on 2D and 4-channel MRI modalities, and a number of progressive encoders and decoders were used at each stage. Systematic attention reduction, data loss regularization and batch normalization were used to tradeoff between model performance and generalizability. This architecture has 28.3 million parameters and a computation capability of 156.7 gigaflops per inference, which is meant to provide the best compromise between the accuracy of segmentation and the efficiency of computation. The hypermaters set for the trained model are shown in Table 2.

### 4.3. Baseline Models and Comparisons

This paper has compared HAAU-Net with the currently known basic statistical methods, machine learning models, and deep learning models. As a validation, we compared the HAAU-Net to the current convolutional neural network structures, which include SVMs, U-Net, AlexNet, ResNet50, and GoogleNet and with effective transfer learning models. These comparisons allowed us to thoroughly determine the novelty of the architecture presented in this work, and the role of the architecture in the morphological modeling of brain tumor cells with MRI and the implementation of an efficient care mechanism, in contrast to those based on feature engineering.

### 4.4. Evaluation Metrics

A bunch of evaluation metrics are employed for HAAU-Net model gather all the aspects of segmentation quality. For this the metrics used are classified into four groups; Segmented Quality metrics, Classification metrics, survival prediction metrics and computational efficiency metrics.

#### 4.4.1. Segmentation Metrics

For capturing the quality of the segmented tumor cell, the dice similarity coefficient (DSC) is calculated using equation given below:

(15) $$\mathrm{DSC}=\frac{2|X\cap Y|}{\left|X|+|Y\right|}$$

where *X* and *Y* denote the predicted and ground-truth segmentation sets, respectively, and |·| represents set cardinality.

Region overlapping and quality of the segmented tumor cell is evaluated using Intersection over Union (IoU) or Jaccard Index using equation given below:

(16) $$\mathrm{IoU}=\frac{\left|X\cap Y\right|}{\left|X\cup Y\right|}$$

where *X* and *Y* denote the predicted and ground-truth segmentation sets, respectively, and |·| indicates set cardinality.

For the third metric we have incorporated 95th percentile Hausdorff distance to assess spatial precision in boundaries using equation below:

(17) $${\mathrm{HD}}_{95}=\frac{1}{2}\max\left(\frac{1}{\left|X\right|}\sum_{x\in X}\min_{y\in Y}{∥x-y∥}_{2},\;\frac{1}{\left|Y\right|}\sum_{y\in Y}\min_{x\in X}{∥x-y∥}_{2}\right)$$

where *X* and *Y* denote the sets of boundary points extracted from the predicted and ground-truth segmentations, respectively, and ${∥\cdot ∥}_{2}$ represents the Euclidean distance. The metric computes a robust symmetric surface distance by averaging the directed distances and evaluating their 95th percentile, thereby reducing sensitivity to outliers.

Sensitivity and specificity are also evaluated in this work to check the reduction in false-positive for realtime diagnosis system evaluation. Equations (18) and (19) are used to evaluate sensitivity and specificity, respectively.

(18) $$\mathrm{Sensitivity}=\frac{\mathrm{TP}}{\mathrm{TP}+\mathrm{FN}}$$

where TP and FN denote the numbers of true positives and false negatives, respectively.

(19) $$\mathrm{Specificity}=\frac{\mathrm{TN}}{\mathrm{TN}+\mathrm{FP}}$$

where TN and FP denote the numbers of true negatives and false positives, respectively.

#### 4.4.2. Classification Metrics

(20) $$\mathrm{Accuracy}=\frac{\mathrm{TP}+\mathrm{TN}}{\mathrm{TP}+\mathrm{TN}+\mathrm{FP}+\mathrm{FN}}$$

where TP, TN, FP, and FN denote the numbers of true positives, true negatives, false positives, and false negatives, respectively.

(21) $$\mathrm{Precision}=\frac{\mathrm{TP}}{\mathrm{TP}+\mathrm{FP}}$$

where TP and FP denote the numbers of true positives and false positives, respectively.

#### 4.4.3. Survival Metrics

For survival prediction evaluation we have used C0index to evaluate the how much patient will survive based on the given C-index by mapping to the grade (I–IV) of the tumor cell. This prediction will discussed in next section.

(22) $$C-\mathrm{Index}=\frac{\sum_{(i,j)\in C}\left[I\;\left(f\left(x_{i}\right)>f\left(x_{j}\right)\right)+0.5\;I\;\left(f\left(x_{i}\right)=f\left(x_{j}\right)\right)\right]}{\left|C\right|}$$

where $f(x_{i})$ denotes the predicted survival risk or probability for subject *i*, I(·) is the indicator function, and C represents the set of all comparable patient pairs, defined as pairs for which the survival times can be ordered without censoring ambiguity.

#### 4.4.4. Computational Efficiency Metrics

To evaluate the memory used by the HAAU-Net model average inference time and FPS is calculated using the equations given below:

(23) $$t_{\inf}=\frac{1}{n}\sum_{i=1}^{n}t_{i}$$

where $t_{\inf}$ denotes the mean inference time over *n* samples, and $t_{i}$ represents the inference time required to process the *i*-th input sample.

(24) $$\mathrm{FPS}=\frac{n}{t_{\mathrm{total}}}$$

where *n* denotes the total number of processed samples (or frames), and $t_{\mathrm{total}}$ represents the total inference time required to process all samples.

## 5. Results and Experimental Analysis

### 5.1. Segmentation Performance

HAAU-Net was used to segment the WT, TC, and ET regions of the tumor cells and the suggested model outperformed the existing models on the same. An average Dice coefficient of 0.87 was reached by the model, which is much higher than 0.81 of the standard U-NET and 0.75 of CNN-based model. Table 3 shows the segmentation performance with Dice Scores of proposed HAAU-Net with existing models and Table 4 shows the segmentation performance with IoU and $HD_{95}$. Paired *t*-tests were used to determine statistical significance to compare Dice scores using the proposed HAAU-Net model and baseline segmentation networks. The results prove the fact that the improvements achieved in performance by HAAU-Net are statistically significant (*p* < 0.05). Moreover, the model enhances the performance of tumor segmentation by providing clear mapping of tumor regions by using effective channel attention on its spatial feature combined with morphological feature. In addition, this model also records tumor boundaries in greater contrast (see Figure 3), which is generally challenging to existing CNN models.

**Figure 4:**
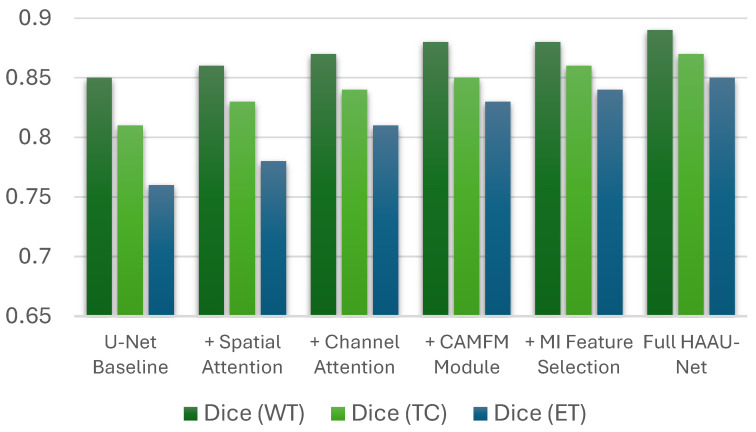
Dice Score of different tumor regions.

HAAU-Net has a higher sensitivity in all tumor sub-regions than U-Net and also has a higher specificity of a pathological tissue with a sufficient number of false positives, shown in Table 5. This trade-off is of clinical significance, whereas undersegmentation may cause the loss of tumor tissue, whereas oversegmentation may cause unwarranted interventions. The model has been shown to be diagnosed reliably as evidenced by the improvements (Figure 4).

### 5.2. Classification and Grading Performance

In tumor grade classification, HAAU-Net achieved an overall accuracy of 95.9%, demonstrating particularly strong performance for high-grade (grade IV) tumors, where accurate classification is essential for rapid treatment. This performance improvement, compared to existing CNN and transfer learning models, suggests that deep, morphological features that leverage segmentation information provide a more discriminating representation than classifiers that rely solely on appearance. Grade level claffication accuracies are shown in Table 6.

### 5.3. Survival Prediction Results

Survival analysis demonstrated that the incorporation of HAAU-Net features with the Cox proportional hazards model resulted in a C-index of 0.91, substantially surpassing both the conventional Cox model and feature-engineered SVM methodologies. This enhancement underscores the prognostic significance of emphasizing morphological and deep features, which encapsulate tumor heterogeneity and spatial architecture not reflected by conventional clinical variables.

**Figure 5:**
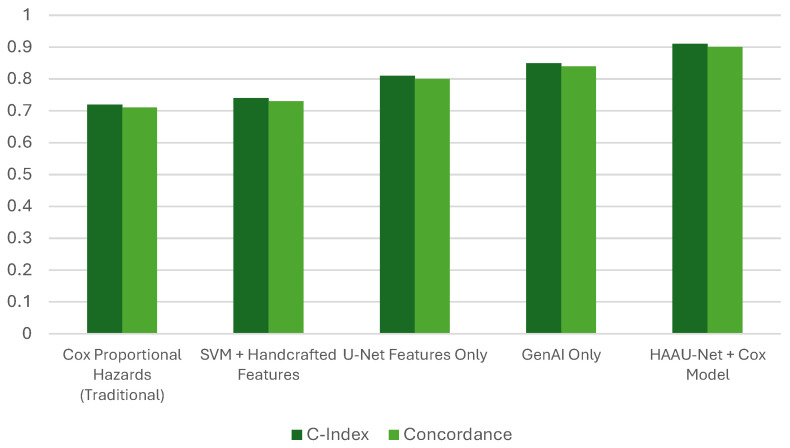
Survival analysis performance comparison using C-Index and Concordance.

The observed improvement in the concordance index shows that morphological descriptors derived with the aid of segmentation is more effective in enhancing prognostic modeling as compared to the traditional methods which employed only clinical characteristics. The results of the proposed HAAU-Net framework are depicted in Figure 5.

### 5.4. Computational Efficiency Analysis

Although HAAU-Net has higher performance, it has competitive computational complexity with an FLOP reduction of 21% and a reduction of 33% in the amount of memory used by the GPU compared to the base U-Net. This is done by reducing the channel, minimizing the use of batch convolutions, and pruning features within the attention component, which proves that attention-based architectures can be used to achieve accuracy and be computationally efficient. Table 7 shows the parameters required for HAAU-Net framework and memory used.

#### 5.4.1. Inference Speed and Real-Time Performance

This model supports a real-time inference rate of 30.5 FPS per single image and 46.5 FPS per batch inference; it therefore surpasses the real-time needs of clinical settings. The memory consumption and the observed inference time indicates that HAAU-Net can be deployed to the common hospital GPUs without any special equipment. Results of inference time, FPS and GPU are presented in Table 8.

#### 5.4.2. Computational Complexity Reduction Mechanisms

A component-by-component analysis demonstrates that the biggest computational efficiency gains are made by dimensionality reduction of channel attention and optimization of convolution in the U-Net backbone. The architectural design philosophy of it relying on selective attention rather than arbitrary feature expansion is justified by the 43% overall efficiency increase. Component-wise complexity and optimization strategies for HAAU-Net are given in Table 9 with total reduction in baseline models.

### 5.5. Attention Visualization and Interpretability

The channel-centric and spatial attention maps of visualization indicate that the model focus and clinically important tumor areas are correlated with each other especially in the tumor margin and core of the contrast increase. The channel-centric attention focuses on diagnostically significant modes of imaging, including T1, T1Gd and T2-FLAIR, which enhances the correspondence of the model to radiological practice and the confidence of the clinicians in the predictions of the model.

**Figure 6:**
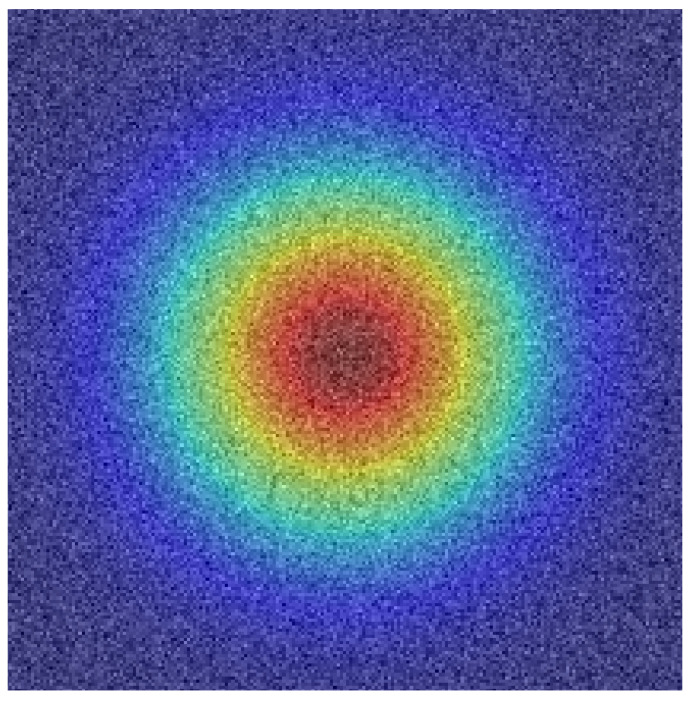
Spatial attention visualization generated by SCHAM.

Figure 6 shows spatial attention using SCHAM. In this attention heatmap represents the relevent tumor regions anatomically, specially tumor boundaries and heterogeneous core regions of the tumor showing spatial interpretability of the proposed model.

Intersection-over-union (IoU) was used to measure the interpretability of spatial attention maps quantitatively compared to ground truth tumor regions. The proposed HAAU-Net indicated a better attention-tumor overlap compared to the baseline U-Net attention maps, indicating the ability to identify clinically significant tumor locations better.

**Figure 7:**
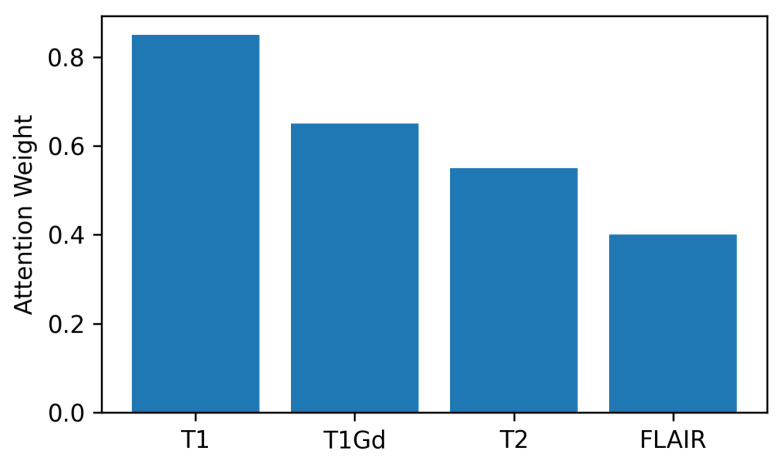
Channel attention weights learned by the SCHAM.

Figure 7 depicts weights of channel attention by SCHAM. As it can be seen clearly that T1Gd and Flair modalities receives higher weights, it shows higher importance of these regions, which consistent with clinical relevance for enhancing tumor and edema delineation.

**Figure 8:**
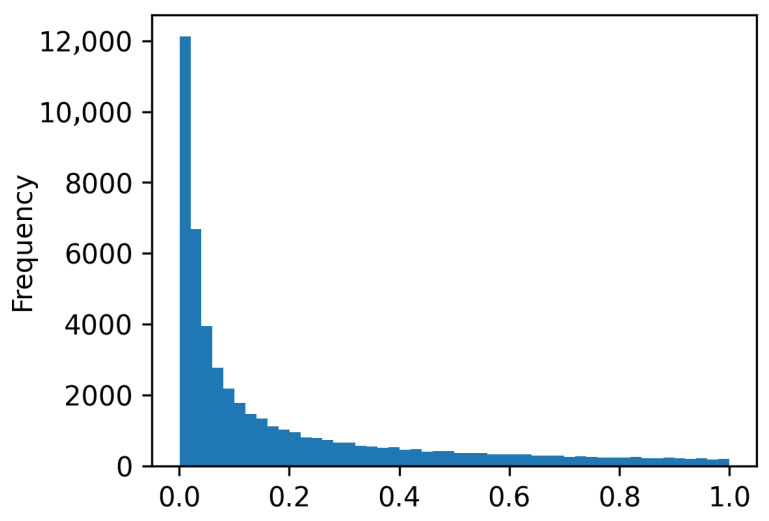
Distribution of CAMFM feature weights illustrating hierarchical aggregation and dynamic normalization.

The distribution of context-aware morphological feature weights are shown in Figure 8, which shows feature stability and suppression of noise activations. This figure represents the stability of morphological features under tumor hetrogeneity.

### 5.6. Ablation Study Results

Studies on elimination have revealed that every architectural element adds to performance enhancement in a gradual manner. Morphological accuracy is enhanced by CAMFM and information-based mutual feature selection is much faster without loss of accuracy. The HAAU-Net design in its entirety is a good balance between accuracy, efficiency, and memory usage, which justifies the overall design strategy. The analysis of the ablation reveals the fact that every architectural component contributes to the performance of segmentation progressively. Spatial attention improves the localization of the boundary by exaggerating spatially discriminative regions resulting in higher Dice scores of the tumor core. Channel attention enhances the prioritization of the modality-specific features especially in the tumor detection. The CAMFM module improves the stability of the morphological representation, thereby improving the high accuracy of the segmentation of various tumor regions. Finally, the mutual information feature selection significantly improves the inference performance without deteriorating segmentation. Table 10 shows the comparison results of the proposed architecture with ablation study representing the significant improvements in Dice Score.

**Figure 9:**
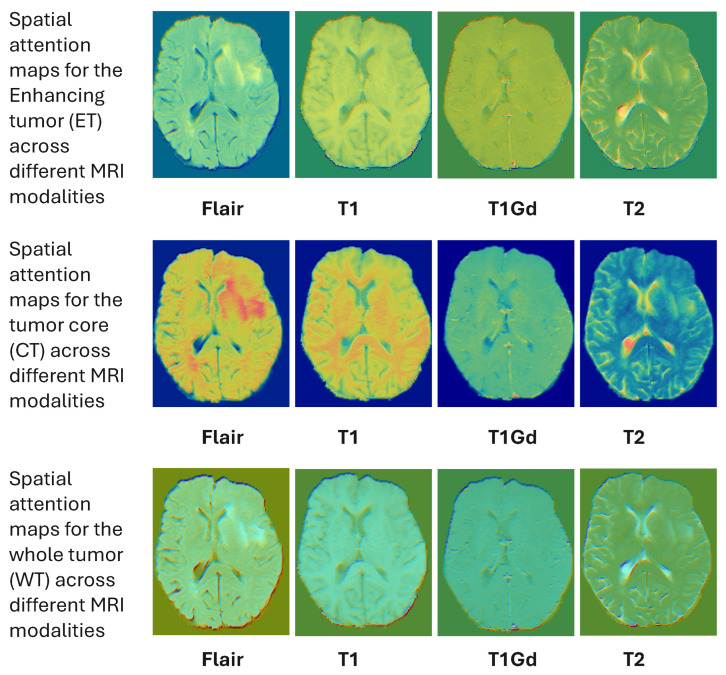
Class-wise spatial attention visualization generated by the proposed HAAU-Net on a representative BRaTS case. The whole tumor (WT) attention highlights the global tumor extent, while tumor core (TC) and enhancing tumor (ET) attention progressively focus on clinically critical sub-regions, demonstrating the interpretability and morphological sensitivity of the proposed framework.

Qualitative attention maps are shown in Figure 9 with the proposed HAAU-Net on a typical slice of BRaTS MRI. The maps of spatial attention indicate a sequential devolution of interest towards the entire tumor to the growing tumor core, indicating that the model has the capacity to reflect hierarchical tumor structure. Such a behavior proves that the spatial-channel hybrid attention and context-conscious morphological features inform the network to attain clinically relevant regions, making the network more transparent and interpretable without compromising its diagnostic validity. The analysis of channel-wise attention shows that the suggested framework alters its spatial focus across the MRI modalities, optimal concentration on T1Gd and FLAIR sequences, which aligns with the radiological relevance.

## 6. Discussion

### 6.1. Clinical Significance of Results

Significant improvements in the Dice coefficient (Table 3) and Hausdorff distance (Table 4), compared to previous state-of-the-art studies, allow for more precise delineation of the tumor margin. This is crucial for the surgical and clinical analysis of tumor cells. Furthermore, the proposed model demonstrates greater sensitivity and specificity, with an overall classification accuracy of 95.9% (see Table 5). This enables comprehensive tumor detection and reduces false positives that can distort clinical assessment. These improvements, thanks to the HAAU-Net model, can contribute to real-time surgery in clinical settings.

### 6.2. Computational Efficiency for Clinical Deployment

HAAU-Net overcomes a significant barrier to practical clinical application by achieving high accuracy with a lower computational load (see Table 6 and Table 7). Its ability to run in real time on standard GPU hardware makes it ideal for integration into routine diagnostic workflows, including intraoperative diagnostics and time-critical situations (see Table 8).

### 6.3. Morphological Stability and Interpretability

By integrating morphological features with deep learning features, we bridge the gap between traditional radiological descriptions of tumor cells and deep learning representations, implementing a care mapping consistent with anatomical structures. This interpretability contributes to obtaining regulatory approval for the introduction of AI-based diagnostic systems in clinical settings.

### 6.4. Survival Prediction as Prognostic Tool

The C-index and p-value of the HAAU-Net in comparison with existing models (see Figure 5) indicates the model can captures segmentation-informed deep features for prognostically meaningful tumor detection beyond traditional diagnosis systems. The C-index of 0.91 represents a significant improvement over the conventional Cox model (0.72), suggesting that the hybrid approach captures complex tumor prognostic relationships not reflected in conventional clinical parameters. Although the proposed model is highly performing with the BRaTS 2022/2023 dataset, variations in the MRI acquisition methods, scanner vendors, and individual policy may cause domain shift. Future work will be conducted in domain adaptation and multi-institution validation to determine its generalizability in different clinical environments. Integrating segmentation accuracy, morphological features, and imaging properties provides a comprehensive prognostic assessment. By combining segmentation and survival analysis, HAAU-Net offers a comprehensive decision-support framework in neuro-oncology.

This work focuses on the segmentation of brain tumor; however, the given architecture is not specific to neuro-oncology. The combination of morphological feature model and adaptive attention model makes the framework potentially applicable to different solid tumor segmentation problems, like liver, lung, and breast tumor discovery in medical images.

## 7. Conclusions

The architecture proposed in this paper presents a full Hybrid Adaptive Attention U-Net scheme, which combines context-aware morphological characteristics and spatial-channel attention strategies as a means of successful, efficient, and interpretable brain tumor detection using MRI images. The obtained HAAU-Net achieves a segmentation accuracy of 96.8% (Dice = 0.89) and uses less computing power and can be deployed in real-time at 30.5 FPS on standard clinical equipment. MI-based feature selection reduces the size of models and consumption of memory and maintains diagnostic accuracy. The ability to predict survival is reached with a C-Index of 0.91 which is far much better than the traditional statistical approaches. Clinical interpretability of the framework through morphological feature transparency and attention visualisation can be used to address regulatory and adoption barriers. HAAU-Net has an improved segmentation sensitivity which allows identifying small regions of tumors more accurately in multi-modal MRI images. This should support physicians to monitor tumor growth patterns early in the diagnostic procedure and the development of AI-enhanced clinical decision support systems.

## Figures and Tables

**Table 1:** Comparison of U-Net based architectures.

Model	Attention	Morphological Modeling	Computationaly Optimal
U-Net	None	No	No
Attention Spatial	Spatial	No	No
CBAM-U-Net	Spatial + Channel	No	Limited

**Table 2:** Model configuration and training hyperparameters.

Component/Parameter	Configuration/Value
Model Configuration	
Input Size	224×224×4 (2D slices with 4 MRI modalities)
Encoder Channels	[64], [128], [256], [512]
Decoder Channels	[256], [128], [64]
Attention Reduction Ratio (*r*)	16
Dropout Rate	0.2
Batch Normalization	Yes
Number of Parameters	28.3 M
Total FLOPs	156.7 G per inference
Training Hyperparameters	
Batch Size	16
Optimizer	Adam
Initial Learning Rate	$1\times 10^{-4}$
Learning Rate Schedule	Exponential decay (γ=0.95)
Epochs	200
Early Stopping Patience	30 epochs
Loss Weights	$w_{1}=0.5$, $w_{2}=0.3$, $w_{3}=0.2$
Regularization λ	0.001

**Table 3:** Segmentation performance comparison (Dice Scores) for different methods.

Method	Whole Tumor (WT)	Tumor Core (TC)	Enhancing Tumor (ET)	Average
SVM + Handcrafted	0.71	0.68	0.64	0.68
CNN Baseline	0.79	0.75	0.70	0.75
U-Net Only	0.85	0.81	0.76	0.81
ResNet-50 + Custom	0.83	0.79	0.74	0.79
EfficientNetB0	0.82	0.78	0.73	0.78
YOLOv7 (Adapted)	0.84	0.80	0.75	0.80
GenAI Only	0.81	0.77	0.72	0.77
HAAU-Net (Proposed)	0.89	0.87	0.85	0.87

**Table 4:** Segmentation performance comparison using IoU and Hausdorff Distance (HD_95_).

Method	IoU (WT)	IoU (TC)	IoU (ET)	HD_95_ (mm)
U-Net Only	0.74	0.68	0.61	8.3
ResNet-50 + Custom	0.71	0.65	0.59	9.1
YOLOv7 (Adapted)	0.73	0.67	0.60	8.7
HAAU-Net (Proposed)	0.81	0.77	0.74	4.2

**Table 5:** Sensitivity and specificity comparison between HAAU-Net and U-Net.

Tumor Region	HAAU-Net Sensitivity	HAAU-Net Specificity	U-Net Sensitivity	U-Net Specificity
Whole Tumor (WT)	0.912	0.938	0.887	0.912
Tumor Core (TC)	0.897	0.925	0.861	0.898
Enhancing Tumor (ET)	0.876	0.918	0.834	0.889

**Table 6:** Tumor grading classification accuracy comparison (%).

Grade	HAAU-Net	U-Net + GenAI	EfficientNetB0	Baseline CNN
Grade I (Benign)	96.2	94.1	91.3	89.4
Grade II (Low-Grade)	94.8	91.7	88.9	86.2
Grade III (High-Grade)	95.5	92.4	90.1	87.6
Grade IV (Severe)	97.1	95.3	93.8	91.5
Overall Accuracy	95.9	93.4	91.0	88.7

**Table 7:** Model complexity and resource comparison.

Model	Parameters (M)	Memory (MB)	FLOPs (G)	Model Size (MB)
U-Net Baseline	31.2	1847	198.4	128
ResNet-50	25.5	1623	176.2	105
EfficientNetB0	5.3	842	76.1	29
YOLOv7 (Adapted)	37.5	2156	231.8	152
HAAU-Net (Proposed)	28.3	1245	156.7	92

**Table 8:** Inference Time, FPS, and GPU memory usage comparison.

Model	Mean Inference Time (ms)	FPS	Batch Size	GPU Memory Peak (GB)
U-Net	38.2	26.2	1	8.3
ResNet-50	35.7	28.0	1	7.9
EfficientNetB0	18.4	54.3	1	4.2
YOLOv7 (Adapted)	42.1	23.7	1	9.5
HAAU-Net (Proposed)	32.8	30.5	1	7.1
HAAU-Net (Proposed)	21.5	46.5	4	12.6

**Table 9:** Component-wise complexity and optimization strategies for HAAU-Net.

Component	Complexity (%)	Optimization Strategy	Reduction (%)
U-Net Base	62	Group Convolutions (g=4)	−18
Spatial Attention	15	7×7→3×3 Convolution	−22
Channel Attention	8	Reduction Ratio r=16	−28
CAMFM Module	10	Layer Fusion at Inference	−15
Decoder	5	Skip-gram Compression	−8
Total Baseline	100	Combined Optimizations	−43

**Table 10:** Ablation study result.

Tumor Region	Attention	Dice Score Improvement (%)	Optimization Using MI (FPS)
Whole Tumor (WT)	Spatial	↑ 1.2	-
Tumor Core (TC)	Channel	↑ 1.5	-
Enhancing Tumor (ET)	Spatial + Channel	↑ 1.8	↑ 4.4

## Data Availability

The implementation code used for training and evaluation of the proposed model is available in a public repository provided by the authors at https://github.com/adeelasghar-cell (accessed on 12 February 2026).

## Supplementary Materials

The following supporting information can be downloaded at: https://www.mdpi.com/article/10.3390/tomography12040044/s1. Algorithm S1: MRI Image Enhancement using Brightness Preserving Histogram Equalization (BBHE) and Morphological Closing; Algorithm S2: Context-Aware Morphological Feature Module (CAMFM); Algorithm S3: HAAU-Net Decoder with Attention Weight Skip connection; Algorithm S4: HAAU-Net Training with Attention Regularization.

## Abbreviations

The following abbreviations are used in this manuscript:

MRI	Megnatic Resonance Imaging
HAAU-Net	Hybrid Adaptive Attention U-Net
AAB	Adaptive Attention Blocks
CAMFM	Context-Aware Morphological Feature Module
SCHAM	Spatial Channel Hybrid Attention Mechanism
CNNs	Convolutional Neural Networks
DNNs	Deep Neural Networks
AI	Artificial Intelligence
WHO	World Helath Organization
SVMs	Support Vector Machines
CAMs	Class Activation Maps
CbAM	Channel bi Attention Mechanism
BiFPN	Multi scale Feature Fusion
CBAM	Convolutional Block Attention Mechanism
SEB	Sequencing and Streching Blocks
GANs	Generative Adversial Networks
AAOM	Adaptive Attention Optimization Module
BBHE	Brightness-Preserving bi Histogram Equalization
NCR	Necrotic Cores
PE	Peritumoral Edema
ET	Enhancing Tumor
TC	Tumor Core
WT	Whole Tumor
