# Supplementary material for: HAAU-Net: Hybrid Adaptive Attention U-Net Integrated with Context-Aware Morphologically Stable Features for Real-Time MRI Brain Tumor Detection and Segmentation
Source: Tomography. 2026 Mar 25;12(4):44. doi: 10.3390/tomography12040044 (PMC13119650; doi:10.3390/tomography12040044)
Supplement: Supplementary file 1 [file tomography-12-00044-s001.zip › tomography-4132796-supplementary.pdf]

Supplementary Materials

Title: **HAAU-Net: Hybrid Adaptive Attention U-Net Integrated with Context-Aware Morphologically Stable Features for Real-Time MRI Brain Tumor Detection and Segmentation.**

This document provides detailed algorithmic descriptions supporting the main manuscript.

Algorithm S1: MRI Image Enhancement using Brightness Preserving Histogram Equalization (BBHE) and Morphological Closing

---

Input: MRI image I with intensity range [0,255]
Output: Enhanced MRI image I_{final}

Step 1: Compute mean gray-scale intensity

$$m \leftarrow mean(I)$$

Step 2: Divide histogram into two sub-histograms

$$H_{low} \leftarrow \{I(i,j) \mid I(i,j) \leq m\}$$
$$H_{high} \leftarrow \{I(i,j) \mid I(i,j) > m\}$$

Step 3: Apply histogram equalization independently:

$$Map\ H_{low}\ from\ [0,m] \rightarrow [0,m]$$
$$Map\ H_{high}\ from\ [m,255] \rightarrow [m,255]$$

Step 4: Combine equalized sub-histograms

$$I_{enhanced} \leftarrow Combine(H_{low\_eq}, H_{high\_eq})$$

Step 5: Apply morphological closing

$$I_{final} \leftarrow MorphClose(I_{enhanced}, kernel=3\times3)$$

Return I_{final}

Algorithm S2: Context-Aware Morphological Feature Module (CAMFM)

Input: Feature map $F \in \mathbb{R}^{\mathbb{h}\times\mathbb{w}\times\mathbb{c}}$
Output: Morphologically stable feature map F_{final}

Step 1: Extract morphological descriptors:
Shape Descriptors:

$$S_{\text{compact}} \leftarrow \frac{4\pi \cdot \text{Area}}{\text{Perimeter}^2} \quad \text{(Compactness)}$$

$$S_{\text{solidity}} \leftarrow \frac{\text{Area}}{\text{ConvexHull\_Area}} \quad \text{(Solidity)}$$

$$S_{\text{circularity}} \leftarrow \frac{\text{Perimeter}^2}{4\pi \cdot \text{Area}} \quad \text{(Circularity)}$$

Texture Features (LBP):

$$T_{\text{contrast}} \leftarrow \sum\left(|\text{grad}_x| + |\text{grad}_y|\right) \quad \text{(Gradient-based contrast)}$$

$$T_{\text{uniformity}} \leftarrow \sum P(i)^2 \quad \text{(Texture uniformity)}$$

Boundary Smoothness:

$$B_{\text{curvature}} \leftarrow \sum \frac{\kappa(s)}{\text{length}} \quad \text{(Mean curvature)}$$

$$B_{\text{regularity}} \leftarrow 1 - B_{\text{roughness}} \quad \text{(Boundary regularity)}$$

Step 2: Hierarchical feature aggregation:
for each scale $s \in \{\text{coarse, intermediate, fine}\}$ **do**
$F_s \leftarrow \text{Conv}_{1\times1}\left(\text{Extract}(F_s)\right)$
$F_s \leftarrow \text{BatchNorm}(F_s)$
end for
Step 3: Compute adaptive weights:
for each morphological descriptor M_i **do**
$w_i \leftarrow \sigma\left(\text{FC}\left(\text{GlobalAvgPool}(F)\right)\right)$ Softmax weight
end for
Step 4: Aggregate morphological descriptors:

$$F_{\text{morph}} \leftarrow \text{LayerNorm}\left(\sum_i w_i \cdot M_i(F)\right)$$

Step 5: Residual integration:

$$F_{\text{final}} \leftarrow F + \text{Dropout}\left(F_{\text{morph}}, p = 0.2\right) \quad \text{(Residual connection)}$$

Return F_{final}

Algorithm S3: HAAU-Net Decoder with Attention Weight Skip connection

Input: Encoder features F_4, Skip connections $\{F_3, F_2, F_1\}$
Output: Segmentation map $S \in \mathbb{R}^{\mathbb{H}\times\mathbb{W}\times 4}$, Attention maps

Step 1: Decoder Level 3:

$F_{4\_up} \leftarrow \text{Upsample}(F_4,\text{scale} = 2)$ {Transposed convolution}

$A_{\text{skip3}} \leftarrow \text{SCHAM}(F_3)$ { Attention-weighted skip connection}

$$F_{\text{concat3}} \leftarrow \text{Concat}\left(F_{4\_up}, A_{\text{skip3}}\right)$$
$$F_{3\_dec} \leftarrow \text{Conv}_{3\times3}(F_{\text{concat3}}, 256 \text{ filters})$$
$$F_{3\_decoded} \leftarrow \text{AAB}\left(F_{3\_dec}\right)$$

Step 2: Decoder Level 2:

$$F_{3\_up} \leftarrow \text{Upsample}\left(F_{3\_decoded},\text{scale} = 2\right)$$
$$A_{\text{skip2}} \leftarrow \text{SCHAM}(F_2)$$
$$F_{\text{concat2}} \leftarrow \text{Concat}\left(F_{3\_up}, A_{\text{skip2}}\right)$$
$$F_{2\_dec} \leftarrow \text{Conv}_{3\times3}(F_{\text{concat2}}, 128 \text{ filters})$$
$$F_{2\_decoded} \leftarrow \text{AAB}\left(F_{2\_dec}\right)$$

Step 3: Decoder Level 1:

$$F_{2\_up} \leftarrow \text{Upsample}\left(F_{2\_decoded},\text{scale} = 2\right)$$
$$A_{\text{skip1}} \leftarrow \text{SCHAM}(F_1)$$
$$F_{\text{concat1}} \leftarrow \text{Concat}\left(F_{2\_up}, A_{\text{skip1}}\right)$$
$$F_{1\_dec} \leftarrow \text{Conv}_{3\times3}(F_{\text{concat1}}, 64 \text{ filters})$$
$$F_{1\_decoded} \leftarrow \text{AAB}\left(F_{1\_dec}\right)$$

Step 4: Output Layer:

$$S_{\text{logits}} \leftarrow \text{Conv}_{1\times1}\left(F_{1\_decoded}, 4 \text{ filters}\right)$$

Four classes (background, ET, ED, NCR)

$$S_{\text{probability}} \leftarrow \text{Softmax}\left(S_{\text{logits}}\right)$$

Probability map per class

Return $S_{\text{probability}}$, Attention maps

Algorithm S4: HAAU-Net Training with Attention Regularization

Require: Training dataset $D = \{(x_i, y_i)\}$, Learning rate α, Epochs E
Hyperparameters: Batch size = 16, Optimizer = Adam ($\beta_1 = 0.9, \beta_2 = 0.999, \epsilon = 10^{-8}$), Learning rate schedule = Exponential decay ($\gamma = 0.95$ per 50 epochs), Early stopping patience = 30 epochs

Step 1: Initialize HAAU-Net weights θ randomly
For epoch $e = 1$ to E **do**
total_loss ← 0, attention_loss ← 0
Shuffle training data D
for each batch $(x_{\text{batch}}, y_{\text{batch}})$ in D **do**
Forward Pass:
$S_{\text{pred}}, A_{\text{spatial}}, A_{\text{channel}} \leftarrow \text{HAAU\_Net}(x_{\text{batch}}; \theta)$
Compute Losses:
$L_{\text{seg}} \leftarrow \text{Hybrid\_Loss}(S_{\text{pred}}, y_{\text{batch}})$
$L_{\text{attn}} \leftarrow \text{Attention\_Regularization}(A_{\text{spatial}}, A_{\text{channel}})$
$L_{\text{batch}} \leftarrow L_{\text{seg}} + L_{\text{attn}}$
Backward Pass: $\nabla_\theta \leftarrow \text{BackProp}(L_{\text{batch}}, \theta)$
Parameter Update: $\theta \leftarrow \theta - \alpha \cdot \nabla\_\theta$
Accumulate Metrics:
$\text{total\_loss} \mathrel{+}= L_{\text{batch}}, \quad \text{attention\_loss} \mathrel{+}= L_{\text{attn}}$
end for
Step 3: Compute Average Metrics
$\text{avg\_loss} \leftarrow \frac{\text{total\_loss}}{\text{num\_batches}} \quad \text{avg\_attn\_loss} \leftarrow \frac{\text{attention\_loss}}{\text{num\_batches}}$
Step 4: Validation: Evaluate on validation set (no gradient updates)
Step 5: Logging
Epoch e: Loss = avg_loss, $Attn\_Loss$ = avg_attn_loss
if validation metric not improving **then**
Apply early stopping
end if
end for
Return trained model θ* = 0
